# Rupture of Didelphys Uterus and Coexistence of Intestinal Diverticulum in an 18 Years Old Primigravida: A Case Report

**DOI:** 10.1002/ccr3.71881

**Published:** 2026-01-15

**Authors:** Asim Ali, Maryam Ilyas, Ali Gohar, Nadeem Yousaf, Masab Ali, Usama Rafiq, Muhammad Husnain Ahmad, Abdul Rehman

**Affiliations:** ^1^ Lahore General Hospital Lahore Punjab Pakistan; ^2^ Guilin Medical University Guilin Guangxi China; ^3^ Punjab Medical College Faislabad Punjab Pakistan; ^4^ St. Tentishev Asian Medical Institute Kant Kyrgyzstan

**Keywords:** disseminated intravascular coagulation, hemoperitoneum, laparotomy, Mullerian duct, obstetric, primigravida, uterine didelphys

## Abstract

Uterine didelphys, also known as double uterus, is a congenital anomaly of the female reproductive system that can be associated with a significant number of threatening obstetric complications, such as uterine rupture. We report a rare and complex case of an 18‐year‐old primigravida who presented with uterine rupture due to uterine didelphys at 18 weeks of gestation. Patient had no prior medical or gynecological history. Physical examination revealed a diffusely distended and tender abdomen along with signs of hemodynamic instability. Ultrasound of the abdomen showed hemoperitoneum, with an intrauterine dead fetus. Second trimester intrauterine rupture was considered the initial diagnosis. After successfully resuscitating the patient, exploratory laparotomy was performed. Uterine didelphys with a right‐sided uterus ruptured from the fundal region along with a wide‐bore intestinal diverticulum was seen. After repairing the uterus in 2 layers and hemodynamically supporting the patient, retained products of conception were removed. Postoperative laboratory investigations were significant for disseminated intravascular coagulation and metabolic acidosis with respiratory compensation. These critical findings necessitated intensive care unit (ICU) admission where the patient received close monitoring, ultimately leading to stabilization of the patient. This case serves as a reminder that adolescents with Mullerian duct anomalies, even without a prior history of uterine scarring, can present with life‐threatening obstetric complications such as uterine rupture. Prompt recognition and timely management are essential to prevent fatal outcomes.

## Key Clinical Message

Early identification of uterine abnormalities through imaging can significantly reduce obstetric complications. Accurate diagnosis enables targeted management, improving pregnancy outcomes. Clinicians should consider uterine evaluation in high‐risk women to optimize reproductive health and minimize risks during pregnancy and childbirth effectively.

## 1 Introduction

Müllerian duct anomalies are congenital defects of the female genital system that arise during the embryonic period, specifically between 6 and 22 weeks of intrauterine life [1]. Embryologically, the Müllerian ducts develop in three stages: organogenesis, fusion, and resorption. During organogenesis, both ducts are formed; failure at this stage results in agenesis or a unicornuate uterus. The next stage, fusion of the ducts, gives rise to the uterus, and incomplete fusion may lead to anomalies such as a bicornuate or didelphys uterus. Finally, failure of resorption of the central septum can result in a septate or arcuate uterus [2]. The link between Müllerian duct anomalies (MDAs) and congenital renal abnormalities arises from their common embryological origin, as both the genital and urinary tracts develop from the urogenital ridges [3].

These anomalies are associated with an increased risk of spontaneous miscarriages, preterm labor, fetal malpresentation, uterine rupture, and a higher incidence of cesarean sections [4, 5, 6]. Approximately 5.5%–6.7% of the female population is affected by Müllerian duct anomalies [4].

Uterus didelphys is a rare form of Müllerian duct anomaly, characterized by the complete failure of fusion of the two Müllerian ducts, resulting in two separate uterine cavities and cervices, also known as a “double uterus” [1]. A longitudinal septum of variable thickness is often associated with septate or didelphys uterus [1, 7]. Furthermore, the development of Müllerian ducts is closely linked to the development of Wolffian ducts, and therefore, renal abnormalities are often found in association with Müllerian duct anomalies. The mean incidence of uterus didelphys among Müllerian duct anomalies is approximately 8.4% [7]. They can also be found incidentally during routine prenatal checkups or in diagnosing the cases of lower abdominal pain as shown in Barut et al. [8].

Uterus didelphys is often asymptomatic, but some patients may present with dyspareunia and dysmenorrhea. Diagnostic investigations include 2D/3D ultrasonography, hysteroscopy, hysterosalpingography, and magnetic resonance imaging (MRI) [1]. Uterine rupture is a rare but catastrophic complication, representing an obstetrical emergency with high fetal and maternal mortality and morbidity. The clinical presentation of a ruptured uterus includes acute abdominal pain and hemodynamic instability. Management involves stabilizing the patient hemodynamically through blood transfusions and fluid resuscitation, followed by exploratory laparotomy and repair of the uterus or hysterectomy [9].

Our case highlights the importance of heightened clinical suspicion and timely intervention to prevent severe morbidity in young patients with congenital uterine anomalies.

## 2 Case History and Examination

An 18‐year‐old Asian female presented to the emergency department with complaints of acute abdominal pain and persistent vomiting for 2 h, alongside a two‐month history of amenorrhea. Notably, she had no prior medical or gynecological history. A bedside ultrasound revealed an unusual finding: an 18‐week intrauterine pregnancy with the fetus displaced into the upper abdominal quadrant, suggestive of a catastrophic intra‐abdominal event. The patient was critically ill on arrival, with pallor, lethargy, hypotension (Blood Pressure 85/60 mmHg), tachycardia (pulse 128 bpm), respiratory distress (Respiratory Rate 28 beats per minute), and oxygen saturation of 81% at room air. Abdominal examination revealed distension, diffuse tenderness, and signs of significant intraperitoneal fluid accumulation.

## 3 Methods (Differential Diagnosis, Investigations, and Treatment)

**FIGURE 1:**
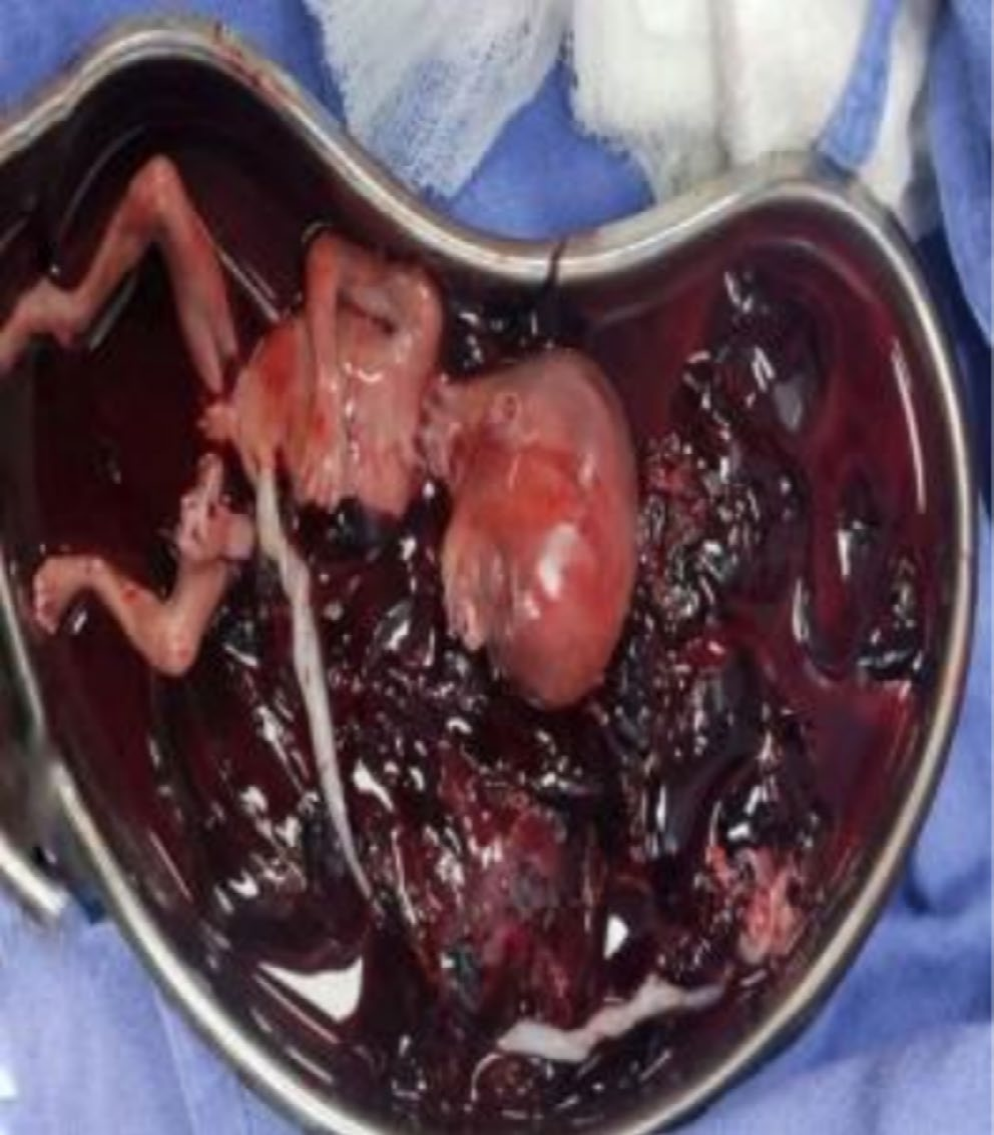
Per‐operative image showing expelled fetus.

**FIGURE 2:**
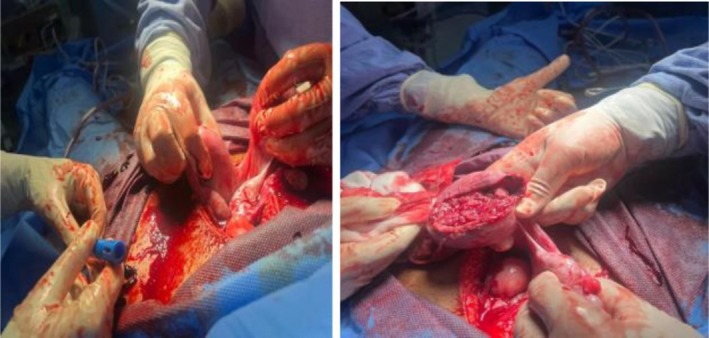
Ruptured uterine tissue.

Emergent ultrasonography of the abdomen and pelvis revealed a massive hemoperitoneum and an intrauterine fetal demise, raising suspicion of uterine rupture. Her diagnosis was further complicated by an unexpected surgical finding: uterine didelphys with rupture of the right‐sided uterus at the fundus and fetal extrusion into the abdominal cavity. Pre‐operatively, the patient suffered cardiac arrest and required 15–20 min of cardiopulmonary resuscitation (CPR) and rapid sequence intubation (RSI). Exploratoration under anesthesia (EUA) was done, which showed that the uterus was 18 weeks in size, the cervical os admitted one finger, the vulva and vagina were healthy, and the bleeding was localized and light. This was followed by surgical exploration which revealed approximately 2000 mL of hemoperitoneum, the expelled fetus (Figure 1), and ruptured uterine tissue (Figure 2). This extremely rare occurrence of second‐trimester uterine rupture in a previously undiagnosed uterine anomaly was compounded by the presence of a wide‐bore intestinal diverticulum, located approximately 2 ft proximal to the ileocecal junction. The presence of the intestinal diverticulum was an incidental finding requiring no intervention, as both the anomalies (uterine and intestinal) originate from different developmental origins. The uterus was repaired in double layers, and retained products of conception were removed and the drain was placed. The patient received aggressive resuscitation with intravenous fluids, 4 units of whole blood, and inotropic support. Postoperative laboratory findings were remarkable for disseminated intravascular coagulation (DIC), with significant thrombocytopenia, prolonged coagulation times, metabolic acidosis, and elevated liver enzymes. The arterial blood gas analysis confirmed severe metabolic acidosis with respiratory compensation.

## 4 Conclusions and Results (Outcome and Follow‐Up)

Following a challenging course of critical care in the Intensive Care Unit (ICU), the patient's condition stabilized, and she was successfully discharged. Timeline summary of events during hospital stay and trends of routine laboratory investigations are mentioned in Tables 1 and 2.

**TABLE 1:** Sequence of events, including the onset of symptoms, diagnosis, surgical interventions, and post‐operative management.

Day	Clinical status	Vital signs	Laboratory results	Diagnostic imaging	Surgical intervention	Postoperative management
Day 1: initial presentation	Vomiting, Abdominal pain	BP = (85/60 mmHg) Pulse = 128 bpm *R*/*R* = gasping SpO_2_ = 81% at room air	Increased white blood cell count Mildly decrease Hb	Abdominal ultrasound: 18 weeks size intrauterine pregnancy and moderate to severe fluid in abdominopelvic cavity	EUA followed by Emergency laparotomy	Intravenous fluids, broad‐spectrum antibiotics, 2FFPs and 2 whole blood transfused Drain was placed
Day 1: postoperative course	Mechanical ventilation Inotropic support (Norepinephrine Infusion)	Vitally stable CNS = sedated DOP = 800 mL since surgery	Stable white blood cell count DIC Metabolic acidosis with respiratory compensation	—	—	Transferred to ICU for close observation
Day 2: ongoing management	Extubated with oxygen mask attached	Tachycardia normotensive (inotropic support tapered) GCS = 14/15 DOP = 100 mL in 12 h	White blood cell count decreasing towards normal	—	—	ICU monitoring and care
Day 3: deterioration	Respiratory distress	*R*/*R* = Tachypnea (39 breaths/min) BP = 110/70 mmHg (Inotropic support stopped) SpO_2_ = 94% with NRM at 10 L oxygen GCS = 15/15 DOP = few mL in last 24 h	Stable white blood cell count	—	—	ICU monitoring and care
Day 6: improvement	Respiratory distress resolved No active complaint	Vitally stable SpO_2_ = 96% at room air GCS = 15/15 DOP = few mL	White blood cell count decreasing towards normal	—	—	Step down to ward
Day 10: discharge	No active complain	Vitally stable GCS = 15/15	White blood cell count decreasing towards normal Normal ABGs Normal clotting profile	—	—	Discharged

Abbreviations: ABGs, arterial blood gases; BP, blood pressure; CNS, central nervous system; DIC, disseminated intravascular coagulopathy; DOP, drain output; EUA, exploration under anesthesia; FFPs, fresh frozen plasma; GCS, glassglow coma scale; Hb, hemoglobin; ICU, intensive care unit; mL, milliliter; NRM, non‐rebreather mask; *R*/*R*, respiratory rate; SpO_2_, oxygen saturation.

**TABLE 2:** Routine laboratory investigations trend during hospital stay.

Parameters	Day 1	Day 3	Day 7	Day 10
Hb (g/dL)	10.7	10.0	11.1	9.65
TLC ×10^3^/μL	24.1	16.2	17.8	13.9
PLT (microliter of blood)	41.8	49	65	85
PT (sec)	17.9	11	11	11
APTT (sec)	48	26	36	30
INR	1.63	1.57	1.3	1.1
Urea (mg/dL)	24	31	35	53
Creatinine (mg/dL)	1.1	1.0	0.8	0.8
Serum sodium (mEql/L)	139	137	138	137
Serum potassium (mEql/L)	5.4	4.8	3.6	4.3
AST (U/L)	141	165	206	95
ALT (U/L)	234	260	313	257
ALP (IU/L)	205	198	155	199
T.Bilirubin (mg/dL)	0.7	0.8	0.8	0.8
Uric acid (mg/dL)	3.8	3.5	3.0	5.9
Calcium (mg/dL)	7.6	7.8	8.0	7.9
Magnesium (mg/dL)	1.18	1.6	2.2	1.12
LDH (U/L)	811	522	366	290
CRP (mg/dL)	64	60	56	54
D‐dimers (ng/mL)	10.3	10.6	11.1	11.5
pH	7.33	7.32	7.41	7.39
pO_2_ (mmHg)	170.1	273.7	198.7	192.1
pCO_2_ (mmHg)	22.0	26.6	32	36
Bicarbonate (mEq/L)	11.8	13.8	16.6	22
Albumin (g/dL)	2.2	2.1	2.1	2.9
Phosphorus (mg/dL)	4.1	4.1	4.3	3.2

## 5 Discussion

This case report presents a diagnostic dilemma involving a uterine anomaly, uterine didelphys, complicated by a 2nd trimester uterine rupture. Interestingly, this case contrasts with previous reports, such as Suthar et al. [9], which suggest that didelphys uterus should be considered in patients with severe dysmenorrhea and chronic pelvic pain. In our case, the patient had no such medical history, highlighting the variable presentation of this uterine anomaly, whose clinical manifestations can range from being symptomatic to asymptomatic. As depicted in Barut et al. [8], the case can be diagnosed incidentally. Spontaneous rupture of a didelphys uterus should be considered as a differential diagnosis in cases of second trimester abdominal pain [1]. However, in cases like ours, where the pregnancy status of the patient is unknown, diagnostic complexity is significantly increased.

Typically, uterine rupture develops in cases where there is a history of uterine scarring as reported by Suthar et al. [9] where the patient had a history of multiple pregnancies and C‐sections before uterine rupture, similarly which contrasts with our case where the patient had no uterine manipulation or manipulation before, which suggests that uterine rupture can also be linked to an unscarred uterus. This highlights a significant knowledge gap between these two conditions [7]. Further research and studies are important to clarify the linkage between uterine rupture and unscarred uterus, enhancing our understanding and management of this life‐threatening complication.

Didelphys uterus has only been reported in previous studies as a part of Herlyn‐Werner‐Wunderlich (HWW) syndrome, also known as obstructed hemivagina and ipsilateral renal anomaly (OHVIRA), which presents as a triad of didelphys uterus, blocked hemi‐vagina, and ipsilateral renal agenesis [10]. The absence of this triad in our case and the presence of a wide‐bore intestinal diverticulum underscore the fact that didelphys uterus might have multiple spectrums of presentation, highlighting the need for more extensive research on this topic. A delayed diagnosis, such as mistaking it for appendicitis [11], can substantially affect patient survival, as timely intervention is pivotal.

This case reinforces the critical need for a multidisciplinary, evidence‐based approach in managing obstetric emergencies associated with congenital uterine anomalies. Early surgical intervention and expert intensive care are instrumental in ensuring a favorable outcome.

The relationship between primary infertility and uterine anomalies is still not fully understood. One proposed explanation is that abnormal uterine morphology may hinder normal fundal implantation, leading instead to implantation along the lateral wall or septum. Such sites often have altered vascular supply and disrupted myometrial or endometrial architecture, which can compromise implantation. In many cases, Müllerian anomalies are incidentally identified during the infertility workup of affected women [12, 13].

Because of the low prevalence of uterus didelphys, data on reproductive outcomes in these patients remain limited and often inconsistent. In a study by Goyal LD ER Al, only one case presented with primary infertility. The impact of a didelphys uterus on fertility therefore remains controversial [14]. Other studies by Nohara et al. [15] and Mashiach et al. [16] reported a case of twin pregnancy in a woman with didelphys uterus and a triplet pregnancy, respectively. On the other hand, a study by Zhang et al. [17] reported that those women with didelphys uterus required infertility treatments more frequently compared to those with other anomalies.

### 5.1 Implication for Practice

Early referral is essential for women with multiple congenital anomalies to rule out Müllerian duct anomalies (MDAs). A comprehensive evaluation at the first pregnancy checkup is particularly important to determine the extent of the condition. Timely recognition and accurate classification enable appropriate management throughout the antepartum, intrapartum, and postpartum periods, thereby optimizing outcomes for both mother and baby [18].

## 6 Conclusion

Patients presenting with uterine tract anomalies, such as uterine didelphys, are at a higher risk of facing obstetric complications. Hence prompt management including surgical intervention should be undertaken without significant delay to prevent mortality. This case report highlights the crucial role of timely intervention, intensive care, and collaborative management in achieving optimal outcomes in these rare and complex cases.

## Author Contributions

**Asim Ali:** conceptualization, writing – original draft, writing – review and editing. **Maryam Ilyas:** conceptualization, writing – original draft, writing – review and editing. **Usama Rafiq:** conceptualization, data curation, writing – original draft, writing – review and editing. **Ali Gohar:** writing – original draft, writing – review and editing. **Abdul Rehman:** conceptualization, writing – original draft, writing – review and editing. **Masab Ali:** conceptualization, writing – original draft, writing – review and editing. **Nadeem Yousaf:** conceptualization, data curation, writing – original draft. **Muhammad Husnain Ahmad:** writing – original draft, writing – review and editing.

## Funding

The authors have nothing to report.

## Ethics Statement

The authors have nothing to report.

## Consent

A written informed consent was obtained from the patient based on the journal's policies.

## Conflicts of Interest

The authors declare no conflicts of interest.

## Data Availability

Data and materials available upon request from corresponding author.
